# Development of a Specific PCR Assay for *Theileria* sp. Yokoyama and Assessment of Its Potential to Cause Anemia in Cattle

**DOI:** 10.3390/pathogens13090735

**Published:** 2024-08-29

**Authors:** Iromy Dhananjani Amarasiri, Kalaichelvan Nizanantha, Ngigi Noel Muthoni Mumbi, Isuru Sachintha Kothalawala, Sampath Madusanka, Wettam Perumage Pavithra Sandamali Indrasiri Perera, Hemal Kothalawala, Thillaiampalam Sivakumar, Naoaki Yokoyama

**Affiliations:** 1 Veterinary Research Institute, Peradeniya 20400, Sri Lanka; 2 Department of Farm Animal Production and Health, Faculty of Veterinary Medicine and Animal Science, University of Peradeniya, Peradeniya 20400, Sri Lanka; 3 National Research Center for Protozoan Diseases, Obihiro University of Agriculture and Veterinary Medicine, Inada-Cho, Obihiro 080-8555, Hokkaido, Japanyokoyama@obihiro.ac.jp (N.Y.); 4 Department of Biochemistry, Faculty of Medicine, University of Peradeniya, Peradeniya 20400, Sri Lanka; 5 National Livestock Development Board, No. 40, Nawala Road, Narahenpita, Colombo 00500, Sri Lanka

**Keywords:** anemia, cattle, PCR, *Theileria* sp. Yokoyama

## Abstract

The clinical implications of *Theileria* sp. Yokoyama, a recently identified *Theileria* species in cattle, remain uncertain. The objective of the present study was to evaluate the anemia status in cattle infected with *Theileria* sp. Yokoyama. Blood samples were collected from 206 cattle across seven Veterinary Ranges in Sri Lanka and analyzed for red blood cell (RBC) indices, including hemoglobin concentration, hematocrit, and RBC counts. Additionally, DNA was extracted from the samples and screened with a newly developed *Theileria* sp. Yokoyama-specific PCR assay targeting the cytochrome b gene. The PCR results revealed that 60 (29.1%) of the surveyed cattle tested positive for *Theileria* sp. Yokoyama, with 47 (78.3%) of them being co-infected with other hemopathogen species. Our findings revealed that the cattle breeds, management systems, and tick infestations are potential risk factors for the *Theileria* sp. Yokoyama infection. Next, we evaluated the anemia status among the surveyed cattle based on the RBC indices. We found that all non-infected cattle were non-anemic. By contrast, anemia was observed in 15 *Theileria* sp. Yokoyama-infected cattle, including 3 singly infected (anemia rate 3/13, 23.1%) and 12 co-infected cattle (12/47, 25.5%). Our findings suggest that *Theileria* sp. Yokoyama causes anemia in infected cattle.

## 1. Introduction

Bovine theileriosis is a disease caused by the species of genus *Theileria* in cattle [1]. Depending on the causative *Theileria* species, bovine theileriosis can be divided into three types: tropical theileriosis caused by *Theileria annulata*, East Coast fever caused by *Theileria parva*, and oriental theileriosis caused by *Theileria orientalis* [1,2]. The lifecycle of *Theileria* species begins with the injection of sporozoites into cattle during the blood-feeding process of infected ticks [3,4]. These sporozoites invade host leukocytes, where they undergo schizogony, forming multinucleated schizonts [5]. The transforming *Theileria* species, namely, *T. parva* and *T. annulata*, induce indefinite proliferation of the leukocytes containing schizonts, leading to severe disease manifestations [6,7]. By contrast, *Theileria orientalis* is a non-transforming *Theileria* species, because the leukocytes containing schizonts of this parasite species do not proliferate [8]. Therefore, as compared to the transforming species, *T. orientalis* typically leads to a milder form of theileriosis [9]. The rupture of schizonts releases merozoites, which infect host red blood cells (RBCs), where they multiply by an asexual reproduction known as merogony [10]. The merozoites released from the infected RBCs invade non-infected RBCs and continue to proliferate by merogony, causing clinical anemia [10,11].

The distribution of *Theileria* species aligns with that of their tick vectors, with *T. annulata* predominantly found in North Africa, Southern Europe, and Asia, while *T. parva* is endemic in Eastern, Central, and Southern Africa [1]. In contrast, *T. orientalis* has global distribution [12]. Sri Lanka, a tropical island nation in the Indian Ocean, harbors a climate conducive to the survival of tick vectors, resulting in the widespread occurrence of tick-borne diseases, including those caused by hemoprotozoan parasites, in cattle populations [13,14,15].

Previous investigations have identified *T. annulata* and *T. orientalis* infections in cattle across Sri Lanka [14,15,16]. However, a recent study aimed at analyzing the genetic diversity of *T. annulata* found that the genetic background of Sri Lankan isolates differed from that of *T. annulata* [17]. In phylogenetic trees constructed with *18S rRNA*, merozoite-piroplasm surface antigen (*tams1*), surface protein (*tasp*), and cytochrome b (*cytB*) gene sequences, the Sri Lankan isolates were not directly related to *T. annulata* but formed a sister clade to the common ancestors of *T. annulata* and *Theileria lestoquardi* [17]. Because of these observations, the *T. annulata*-like species discovered in Sri Lanka is now considered a distinct parasite species and has been provisionally designated as *Theileria* sp. Yokoyama [17]. Recent studies detected this potentially novel parasite species in cattle in India and camels in Egypt [18,19]. Despite these advancements, the clinical significance of *Theileria* sp. Yokoyama remains uncertain due to the lack of specific diagnostic tests, particularly PCR assays. The *tams1*-like and *tasp*-like genes of *Theileria* sp. Yokoyama encode surface proteins, which are usually diverse among field isolates [20]. The *18S rRNA* may not be an ideal candidate for developing PCR assays due to its high degree of similarity among different *Theileria* species [1,17]. On the other hand, the *cytB* gene in *Theileria* sp. Yokoyama is highly conserved but differs from similarly conserved *T. annulata cytB* gene sequences [17]. Importantly, the *cytB* gene of *Theileria* sp. Yokoyama contains 122 unique single nucleotide polymorphisms compared to that of *T. annulata* [17]. Thus, validation of PCR results through sequencing and phylogenetic analyses is straightforward [21]. Consequently, the present study aimed to develop a *cytB*-based PCR assay for *Theileria* sp. Yokoyama and employ it to assess the clinical relevance of *Theileria* sp. Yokoyama infection in a cross-sectional cattle survey.

## 2. Materials and Methods

### 2.1. Development of a PCR Assay Specific for Theileria sp. Yokoyama

**Figure 1:**
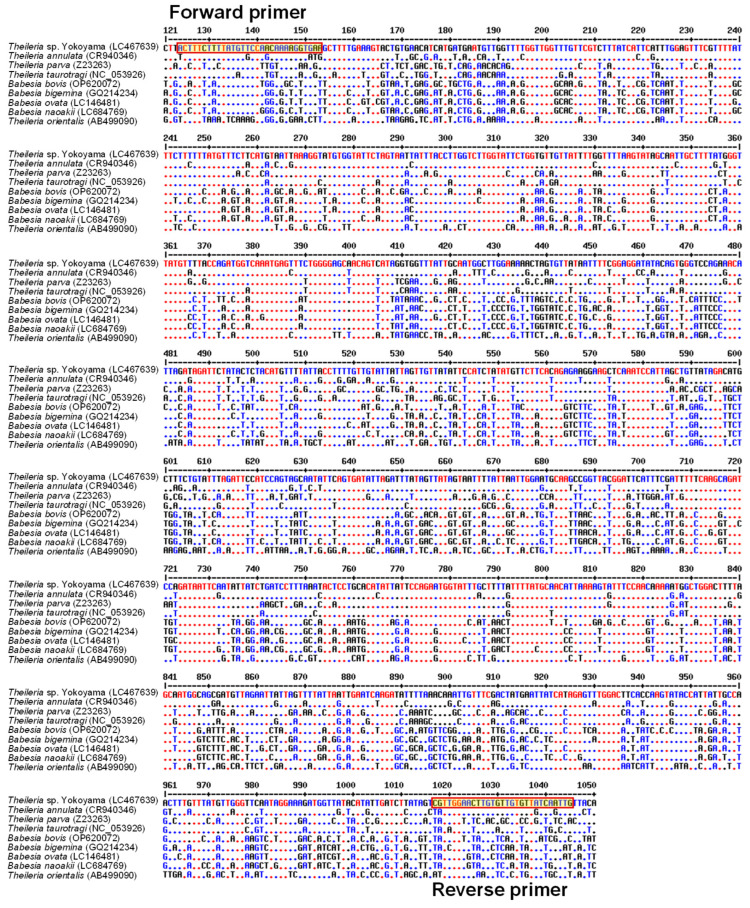
Designing primers. *Theileria* sp. Yokoyama-specific forward and reverse primers were designed based on the alignment of cytochrome b gene sequences from various bovine *Theileria* and *Babesia* species. Boxed regions indicate the target nucleotide sequences of the forward and reverse primers. Dots denote nucleotides that are identical to the *Theileria* sp. Yokoyama sequence.

A PCR assay specific to *Theileria* sp. Yokoyama was developed, targeting the *cytB* gene. Following a multiple alignment of *cytB* gene sequences of various bovine hemopathogens, a set of forward (5′-ACTTTCTTTTATGTTCCAACAAAAGGTGAA-3′) and reverse (5′-CAATTGATAACACAACACAAGTTCCAACG-3′) primers specific to *Theileria* sp. Yokoyama were designed (Figure 1). The specificity of the PCR assay was evaluated using a panel of DNA samples from bovine *Theileria* (*Theileria* sp. Yokoyama, *T. annulata*, *T. parva*, and *T. orientalis*), *Babesia* (*Babesia bovis*, *Babesia bigemina*, *Babesia ovata*, and *Babesia naoakii*), *Trypanosoma* (*Trypanosoma evansi*, *Trypanosoma brucei*, and *Trypanosoma theileri*), and *Anaplasma* (*Anaplasma marginale* and *Anaplasma centrale*) species [17,22]. PCR reactions were performed in a 10 μL reaction mixture containing 1 μL of 10× PCR buffer (Applied Biosystems, Branchburg, NJ, USA), 1 μL of 2 mM dNTP mix (Applied Biosystems), 0.5 μL of 10 μM each of forward and reverse primers, 0.1 μL of AmpliTaq Gold DNA polymerase (Applied Biosystems), 1 μL of template DNA, and 5.9 μL of distilled water. The cycling conditions comprised an initial denaturation at 95 °C for 5 min, followed by 45 cycles of denaturation at 95 °C for 30 s, annealing at 60 °C for 1 min, extension at 72 °C for 2 min, and a final elongation at 72 °C for 7 min. PCR products were analyzed by agarose gel electrophoresis, and the detection of a 922 bp amplicon indicated a positive result for *Theileria* sp. Yokoyama infection. The sensitivity of the PCR assay was evaluated using a pCR2.1 plasmid containing the full-length *cytB* gene of *Theileria* sp. Yokoyama (GenBank accession number LC467667) from a previous study [17]. Briefly, the plasmid DNA sample was diluted using distilled water to obtain concentrations of 100, 50, 25, 10, 5, 2, and 1 copies of plasmids per microliter. Subsequently, one microliter of each dilution was subjected to *cytB* PCR, as described above.

### 2.2. Blood Sampling

A cross-sectional survey was conducted to assess the clinical significance of *Theileria* sp. Yokoyama infection in cattle in Sri Lanka using the developed PCR assay. Blood samples were collected from a total of 206 apparently healthy cattle across several farms in seven veterinary ranges, including six ranges in the Polonnaruwa district (Polonnaruwa, Hingurakgoda, Bakamuna, Medirigiriya, Aralaganwila, and Welikanda) and one (Melsiripura) in the Kurunegala district. The following data were recorded for each animal: sex, age, breed, grazing management system (intensive, semi-intensive, and extensive), and presence of ticks. From each animal, 2 mL of blood sample were collected from the jugular vein into an EDTA-containing vacutainer tube.

### 2.3. Measurement of RBC Indices

Within 24 h of sampling, each blood sample was analyzed for RBC indices, including hemoglobin concentration (Hb), hematocrit (HCT), and RBC counts. Using HemoCue Hb 201 (Ängelholm, Skåne, Sweden), Hb was measured according to the manufacturer’s instructions. On the other hand, using manual methods, HCT [23] and RBC counts [24] were determined. Anemia was defined as concurrent reductions in Hb, HCT, and RBC counts below 24%, 8 g/dL, and 5 × 10^6^/μL, respectively [25].

### 2.4. Microscopic Detection of Theileria, Babesia, and Anaplasma Species

Thin blood smears were prepared from the collected blood samples, stained with Leishman, and then observed under a light microscope for detecting hemopathogens, including species of *Theileria*, *Babesia*, and *Anaplasma* [26,27].

### 2.5. PCR Detection of Theileria sp. Yokoyama and Other Bovine Hemopathogens

DNA was extracted from each blood sample using the QIAamp DNA Blood Mini Kit (Qiagen, Hilden, Germany) following the manufacturer’s instructions and then stored at −20 °C until PCR screening. All 206 cattle DNA samples were screened for *Theileria* sp. Yokoyama infection, as described above. Additionally, these DNA samples were screened for other bovine hemopathogens, including *T. orientalis*, *B. bovis*, *B. bigemina*, *B. naoakii*, and *A. marginale*, using previously described PCR assays [22]. In each PCR assay, a DNA sample of the target hemopathogen species and a no-template reaction mixture were used as positive and negative controls, respectively.

### 2.6. Sequencing and Phylogenetic Analyses

Randomly selected amplicons from *Theileria* sp. Yokoyama-specific PCR assay were gel extracted, cloned, and sequenced as previously described [14]. The resultant gene sequences and previously reported *cytB* gene sequences representing *Theileria* sp. Yokoyama, *T. annulata*, *T. lestoquardi*, *T. parva*, *Theileria taurotragi*, *T. orientalis*, and *Babesia duncani* (outgroup) were aligned using MAFFT online software version 7 (https://mafft.cbrc.jp/alignment/server/index.html, accessed on 19 July 2024) [28]. The alignment was then analyzed with MEGA version 11 software to predict the best substitution model based on the lowest Akaike Information Criterion value [29]. A maximum likelihood phylogenetic tree was then constructed based on the Hasegawa–Kishino–Yano substitution model (G + I) using MEGA [30,31].

### 2.7. Statistical Analyses

*p*-Values were calculated using an “N-1” chi-squared test (https://www.medcalc.org/calc/comparison_of_proportions.php, accessed on 10 July 2024) to assess significant variations in positive rates across various groups of surveyed cattle [32,33]. The differences between the positive rates were considered significant if the *p*-values were less than 0.05.

## 3. Results

### 3.1. Development of a Theileria sp. Yokoyama-Specific PCR Assay

**Figure 2:**
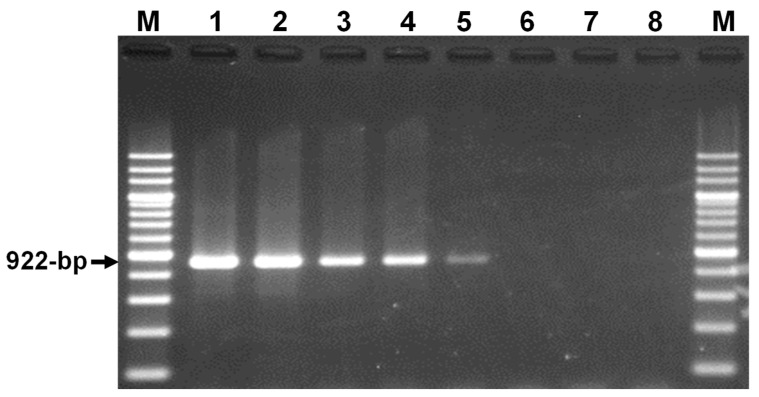
Sensitivity testing. The sensitivity of the newly developed PCR assay was tested by using a plasmid DNA with an insert of the full-length cytochrome b gene (*cytB*) from *Theileria* sp. Yokoyama. Lanes 1–7 represent the use of 100, 50, 25, 10, 5, 2, and 1 copies of plasmid, respectively, as templates. Lane 8 indicates a no-template negative control, while M denotes a 200 bp DNA ladder marker. The PCR assay was able to detect as few as five copies of *cytB*.

The present study successfully developed a PCR assay targeting the *cytB* gene for detecting *Theileria* sp. Yokoyama infection. The PCR assay was highly specific in detecting *Theileria* sp. Yokoyama, as it did not produce any amplicons when DNA samples from several other bovine hemopathogens, including species of *Theileria*, *Babesia*, and *Anaplasma*, and non-infected cattle, were used as templates. In addition, the high sensitivity of the PCR was evident, as it detected as few as five copies of *Theileria* sp. Yokoyama *cytB* gene (Figure 2).

### 3.2. Microscopic and PCR Detection of Theileria sp. Yokoyama and Other Bovine Hemopathogens

Microscopic analysis of thin blood smears revealed that the surveyed cattle were infected with species of *Theileria*, *Babesia*, and *Anaplasma*. Of 206 cattle surveyed, 110 (53.3%), 17 (8.3%), and 88 (42.7%) were microscopy positive for *Theileria*, *Babesia*, and *Anaplasma* species, respectively (Table 1). *Theileria* and *Anaplasma* species were detected in all surveyed veterinary ranges, whereas *Babesia* species were detected in cattle from five veterinary ranges, excluding Hingurakgoda and Melsiripura.

**Figure 3:**
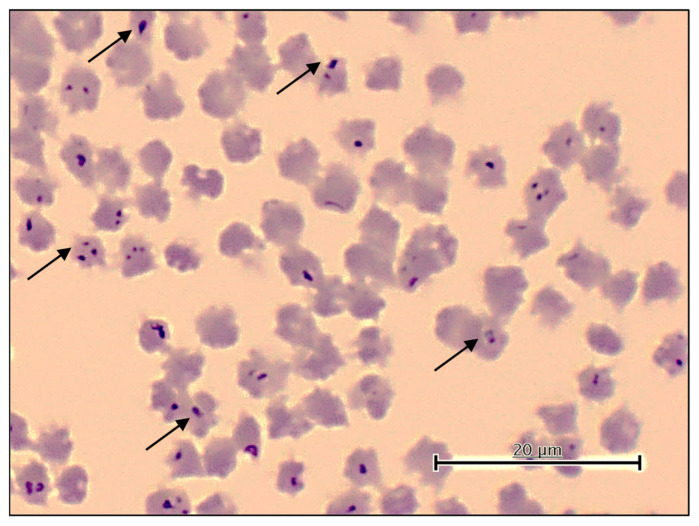
Microscopic image showing *Theileria* sp. Yokoyama within red blood cells (indicated by arrows).

Considering the limitations of microscopy in sensitivity and species differentiation, the newly developed *cytB*-based PCR assay was employed to detect *Theileria* sp. Yokoyama, alongside previously described PCR assays for other pathogens. *Theileria* sp. Yokoyama was detected in 60 (29.1%) samples, while *T. orientalis*, *B. bovis*, *B. bigemina*, and *A. marginale* were detected in 98 (47.6%), 8 (3.9%), 14 (6.8%), and 128 (62.1%) of the samples, respectively (Table 1). All surveyed cattle were, however, negative for *B. naoakii* infection. All microscopy-positive samples were confirmed to be positive in the respective PCR assays. *Theileria* sp. Yokoyama was detected in all surveyed veterinary ranges, with positive rates ranging from 9.8% to 63.9% (Table 1). The microscopic examination of blood smears obtained from cattle singly infected with *Theileria* sp. Yokoyama, as indicated by PCR results, revealed the presence of this parasite species within RBCs. In particular, one of these cattle had high parasitemia and was anemic, as evidenced by lower values of Hb, HCT, and RBC counts compared to the lower limit of the normal range (Figure 3, Table 2) [25].

**Figure 4:**
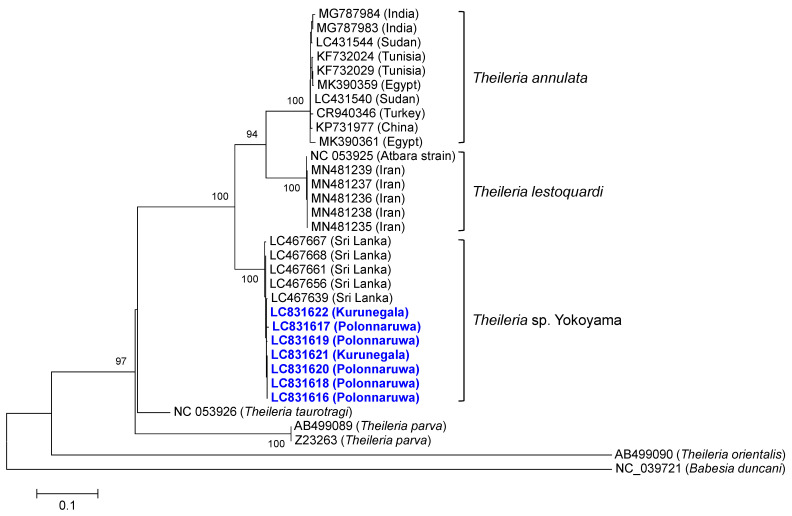
Phylogenetic analysis of cytochrome b (*cytB*) gene sequences. The *cytB* sequences determined from the amplicons derived from *Theileria* sp. Yokoyama PCR assay and those from *Theileria* sp. Yokoyama, *Theileria annulata*, *T. lestoquardi*, *T. parva*, *T. taurotragi*, and *T. orientalis*, which had been previously registered with the GenBank, were used to construct a maximum likelihood phylogenetic tree. A *Babesia duncani* sequence was used as an outgroup. The newly determined sequences (highlighted in blue) clustered together with previously reported *Theileria* sp. Yokoyama sequences and formed a sister clade to the common ancestor of *T. annulata* and *T. lestoquardi*.

The sequences (GenBank accession numbers: LC831616–LC831622) of seven amplicons from *Theileria* sp. Yokoyama-specific PCR assay shared 99.57–99.89% identity scores with previously reported *cytB* gene sequences of *Theileria* sp. Yokoyama (LC467639, LC467640, LC467661, and LC467663) and clustered together in phylogeny (Figure 4). The *Theileria* sp. Yokoyama clade formed a sister clade to the common ancestor of *T. annulata* and *T. lestoquardi* sequences.

### 3.3. Potential Risk Factors for Theileria sp. Yokoyama Infection

Analysis of *Theileria* sp. Yokoyama-positive rates in relation to sex, age groups, cattle breed, grazing management practices, and tick infestation status revealed correlations with breed, management practices, and tick infestations. The positive rates were significantly higher in *Bos taurus* and cross-bred cattle than in *Bos indicus* cattle (Table 3). Cattle maintained under extensive grazing systems had higher positive rates than those under intensive and semi-intensive systems. Additionally, the positive rate was higher in cattle with tick infestations as compared to those without (Table 3).

### 3.4. Anemia Status of Theileria sp. Yokoyama-Infected Cattle

Anemia rates were analyzed based on Hb, HCT, and RBC counts among different categories of cattle according to their infection status. Of the surveyed cattle, 177 (85.9%) cattle were positive for at least one pathogen species, and 29 (14.1%) were negative for all of the surveyed pathogens (Table 4). Among the infected cattle, 102 (57.6%) were co-infected with two, three, or four pathogens. Of the 60 cattle infected with *Theileria* sp. Yokoyama, 13 (21.7%) were singly infected and 47 (78.3%) were co-infected with other hemopathogen species (Table 4). None of the non-infected cattle were anemic, whereas 31 (17.5%) of the 177 infected animals were anemic. Within the group of anemic animals, *Theileria* sp. Yokoyama infection was detected in 15 (48.4%) cattle, including 3 cattle that were singly infected, as well as 6, 5, and 1 cattle that were co-infected with *A. marginale*, *T. orientalis* and *A. marginale*, and *B. bovis*, *B. bigemina*, and *A. marginale*, respectively, along with *Theileria* sp. Yokoyama (Table 4).

## 4. Discussion

The present study addressed two crucial aspects of *Theileria* sp. Yokoyama: the development of a diagnostic PCR assay and an assessment of its clinical impact on infected cattle. The *cytB*-based PCR assay developed in this study was highly specific to *Theileria* sp. Yokoyama, as confirmed through specificity testing and field evaluation followed by sequencing and phylogenetic analyses. In general, a PCR assay for *Theileria* species is considered highly sensitive if it can detect 3–10 copies of the template DNA [1]. Therefore, the PCR assay developed in this study can be regarded as highly sensitive, as it could detect as few as five copies of the *Theileria* sp. Yokoyama *cytB* gene. These findings suggest that the newly developed *cytB* PCR assay can be conveniently used to detect *Theileria* sp. Yokoyama infection for various purposes, including surveillance and diagnosis.

Using the newly developed PCR assay, we observed that *Theileria* sp. Yokoyama infection is common among cattle in Sri Lanka. Next, we investigated whether the infection rate is associated with sexes, age groups, breeds, grazing management practices, or tick infestations. Our findings revealed higher infection rates in *B. taurus* and cross-bred cattle than in *B. indicus*, in cattle managed under extensive systems versus those managed by intensive or semi-intensive systems, and in cattle with tick infestations as opposed to those without. These results are unsurprising given that *Theileria* species are transmitted by ticks, which have a greater chance of infesting freely grazing cattle under extensive management practices. Furthermore, *B. indicus* cattle are more genetically resistant against tick infestations compared to *B. taurus* and their crosses [34]. Additionally, *B. indicus* cattle may have developed a stronger immunity, leading to parasite clearance or very low parasitemia [35]. These factors could explain the higher rate of *Theileria* sp. Yokoyama infection in *B. taurus* or cross-bred cattle than in *B. indicus*.

We found that anemia was common in cattle infected with the hemopathogens surveyed in this study, but not in those that tested negative. Although anemia can also be caused by other factors, such as gastrointestinal parasites, ticks, and non-infectious causes [36], this possibility can be ruled out since all animals negative for the surveyed hemopathogens were non-anemic. Among the hemopathogens surveyed in the present study, *T. orientalis*, *B. bovis*, *B. bigemina*, and *A. marginale* are well known for their ability to cause anemia [9,22,27]. However, it was previously unclear whether *Theileria* sp. Yokoyama could induce anemia in cattle. In the present study, anemia was observed in a quarter of the cattle infected with *Theileria* sp. Yokoyama, including 3 out of 13 animals with single infection, suggesting that this pathogen may have the potential to cause anemia in cattle. Anemia in *Theileria*-infected cattle arises from the asexual reproduction of merozoites, known as merogony, within the infected RBCs [10]. Anemia is a frequent clinical sign of bovine theileriosis caused by *T. annulata* but not by *T. parva* [10]. These observations suggest that the merogony of *Theileria* sp. Yokoyama might resemble that of *T. annulata*. The high rate of anemia in cattle infected with *Theileria* sp. Yokoyama may lead to significant economic consequences, emphasizing the importance of control measures.

The clinical presentation of acute theileriosis may vary based on the causative *Theileria* species. During acute infection, anemia is the primary clinical sign in cattle infected with *T. orientalis*, a non-transforming *Theileria* species, whereas in cattle infected with *T. annulata* and *T. parva*, clinical signs are associated with lymphoproliferation [37,38]. Phylogenetic trees show that *Theileria* sp. Yokoyama shares a common ancestor with known transforming *Theileria* species, suggesting that *Theileria* sp. Yokoyama may also have the ability to transform the infected lymphocytes [17]. However, additional investigations into the acute phase of theileriosis caused by *Theileria* sp. Yokoyama, along with the in vitro cultivation of its schizonts, are necessary to confirm this assumption.

## 5. Conclusions

Our study demonstrates that *Theileria* sp. Yokoyama causes anemia in infected cattle, potentially leading to considerable economic losses to the cattle industry. Further research is essential to fully understand the clinical manifestations and pathogenesis of *Theileria* sp. Yokoyama infection for developing effective disease management strategies.

## Figures and Tables

**Table 1:** A summary of microscopy and PCR results.

District	Veterinary Range	No. Animals	No. Microscopy Positive (%)			No. PCR Positive (%)				
			*Theileria*	*Babesia*	*Anaplasma*	*Theileria* sp. Yokoyama	*T. orientalis*	*B. bovis*	*B. bigemina*	*A. marginale*
Polonnaruwa	Medirigiriya	21	9 (51.2)	2 (23.8)	8 (38.0)	5 (23.8)	8 (38.1)	5 (23.8)	3 (14.3)	10 (47.6)
	Polonnaruwa	36	29 (80.5)	5 (13.8)	18 (50.0)	23 (63.9)	14 (38.9)	1 (2.8)	5 (13.9)	24 (66.7)
	Hingurakgoda	20	11(55.0)	0 (0.0)	3 (30.0)	6 (30.0)	11 (55.0)	0 (0.0)	0 (0.0)	9 (45.0)
	Welikanda	30	12 (40.0)	1 (3.3)	12 (40.0)	3 (10.0)	13 (43.3)	0 (0.0)	1 (3.3)	16 (53.3)
	Bakamuna	41	21(63.4)	3 (7.3)	15 (63.4)	4 (9.8)	23 (56.1)	2 (4.9)	2 (4.9)	26 (63.4)
	Aralaganwila	34	15 (44.1)	3 (8.8)	15 (44.2)	4 (11.8)	15 (44.1)	0 (0.0)	3 (8.8)	19 (55.9)
Kurunagela	Melsiripura	24	13 (54.2)	0 (0.0)	14 (58.3)	15 (62.5)	14 (58.3)	0 (0.0)	0 (0.0)	24 (100)
Total		206	110 (53.3)	17 (8.3)	88 (42.7)	60 (29.1)	98 (47.6)	8 (3.9)	14 (6.8)	128 (62.1)

**Table 2:** Characteristic of a cow that was singly infected with *Theileria* sp. Yokoyama and had high parasitemia.

Breed	Jersey
Age	3.5 years
Physiological status	Pregnant
Management	Extensive
Tick infestation	Yes
Rectal temperature	38.8 °C
Hemoglobin concentration	7.1 g/dL
Hematocrit	21%
RBC counts	3.32 × 10^6^/μL
Parasitemia	7.8%

**Table 3:** Positive rates of *Theileria* sp. Yokoyama in relation to potential risk factors.

Risk Factors	No. Animals	No. Positive (%)	*p*-Value
Sex			
Female	188	54 (28.7)	0.6822
Male	18	6 (33.3)	
Age			
1–3 years	165	47(28.48)	0.6854
>3 years	41	13 (31.7)	
Breed ^a^			
*Bos taurus*	6	3 (50)	0.0473 (Bt vs. Bi)
*Bos indicus*	72	12 (16.6)	0.0054 (Bi vs. Cr)
Cross-bred	128	45 (35.2)	0.4601 (Bt vs. Cr)
Management ^b^			
Extensive	93	59 (63.4)	<0.0001 (Ex vs. In)
Intensive	24	0 (0)	0.6041 (In vs. Si)
Semi-intensive	89	1 (1.1)	<0.0001 (Ex vs. Si)
Ticks			
Presence	132	53 (40.2)	<0.0001
Absence	74	7 (9.5)	

^a^ Bt, *B. taurus*; Bi, *B. indicus*; Cr, cross-bred; ^b^ Ex, extensive; In, intensive; Si, semi-intensive.

**Table 4:** Anemia status in cattle with single and co-infections involving *Theileria* sp. Yokoyama.

Pathogen	No. Animals (% ^a^)	No. Anemic Animals (% ^b^)
Non-infected	29 (14.1)	0 (0.0)
Single pathogen		
*Theileria* sp. Yokoyama	13 (6.3)	3 (23.1)
*T. orientalis*	23 (11.2)	3 (13.0)
*B. bovis*	1 (0.5)	0 (0.0)
*B. bigemina*	4 (1.9)	1 (25.0)
*A. marginale*	34 (16.5)	0 (0.0)
Two pathogens		
*Theileria* sp. Yokoyama + *T. orientalis*	5 (2.4)	0 (0.0)
*Theileria* sp. Yokoyama + *A. marginale*	18 (8.7)	6 (33.3)
*T. orientalis* + *B. bovis*	1 (0.5)	0 (0.0)
*T. orientalis* + *B. bigemina*	2 (1.0)	0 (0.0)
*T. orientalis* + *A. marginale*	47 (22.8)	10 (21.3)
*B. bovis* + *A. marginale*	1 (0.5)	0 (0.0)
*B. bigemina* + *A. marginale*	2 (1.0)	2 (100)
Three pathogens		
*Theileria* sp. Yokoyama + *T. orientalis* + *A. marginale*	19 (9.2)	5 (26.3)
*Theileria* sp. Yokoyama + *B. bovis* + *A. marginale*	1 (0.5)	0 (0.0)
*Theileria* sp. Yokoyama + *B. bigemina* + *A. marginale*	2 (1.0)	0 (0.0)
*B. bovis* + *B. bigemina* + *A. marginale*	1 (0.5)	0 (0.0)
Four pathogens		
*Theileria* sp. Yokoyama + *B. bovis* + *B. bigemina* + *A. marginale*	2 (1.0)	1 (50.0)
*T. orientalis* + *B. bovis* + *B. bigemina* + *A. marginale*	1 (0.5)	0 (0.0)
Total	206	31 (15.0)

^a^ Expressed as a percentage of total number of animals (*n* = 206). ^b^ Expressed as a percentage of number of animals in each infection status category.

## Data Availability

All data generated in this study were included in this article.
